# Geographic pattern of asthma prevalence in Brazilian adolescents: a systematic review with meta-analysis

**DOI:** 10.1016/j.jped.2024.12.004

**Published:** 2025-02-14

**Authors:** Marcela Claudia de Paula Oliveira, Emília Chagas Costa, Vanessa Sá Leal, Estefany Karolayne dos Santos Machado, Emanuel Sávio Cavalcanti Sarinho, Ricardo de Freitas Dias, Risia Cristina Egito de Menezes, Mauro Virgílio Gomes de Barros, Ricardo Almendra, Marco Aurélio de Valois Correia Junior

**Affiliations:** a Universidade de Pernambuco (UPE), Programa de Pós-Graduação em Hebiatria, Recife, PE, Brazil; b Universidade Federal de Pernambuco (UFPE), Programa de Pós-Graduação em Nutrição, Recife, PE, Brazil; c Universidade Federal de Pernambuco (UFPE), Hospital das Clínicas, Departamento de Imunoalergologia, Recife, PE, Brazil; d Universidade Federal de Alagoas (UFAL), Faculdade de Nutrição, Maceió, AL, Brazil; e University of Pernambuco and Federal University of Paraiba Associate Postgraduate Program in Physical Education, Recife, Pernambuco, Brazil; f University of Coimbra, Department of Geography and Tourism and Centre of Studies in Geography and Spatial Planning (CEGOT), Coimbra, Portugal; g University of Coimbra, Department of Geography and Tourism, Coimbra, Portugal

**Keywords:** Asthma, Asthma prevalence, Adolescents, Brazil, Geography and health

## Abstract

**Objectives:**

This study aimed to verify the prevalence of asthma in Brazilian adolescents and its geographic pattern carried through a systematic review and meta-analysis.

**Sources:**

A survey of databases (Pubmed, Chocrane, LILACS, SCIELO and SCOPUS) was carried out, following the PRISMA statement, between the years 2013 and 2023 (PROSPERO-ID-CRD42023427988). Articles that presented a measure relative to the prevalence of asthma in adolescents were included. The methodological quality regarding risk of bias was assessed according to the approach proposed by the Joanna Brigg Institute.

**Summary of the findings:**

Ten of a total of 3140 studies were included. Six studies were collected before 2015. The prevalence of active asthma, severe asthma, and diagnosed asthma were 18 %, 6 %, and 14 %, respectively. The studies were presented in large urban centers and capitals, had a high methodological quality, and showed high heterogeneity. Subgroup analysis was carried out, separating the cities according to the different regions of Brazil (north, northeast, center-west, south, and southeast). The northeast was the region with the lowest prevalence of active asthma (14 %, 95 % CI = 12–17 %, *p* < 0.001). There was no difference between regions in terms of diagnosed asthma and severity.

**Conclusion:**

A high proportion of Brazilian adolescents reported having active asthma, with the northeast region having the lowest prevalence. Most of the studies were collected more than eight years ago and regard capitals and large urban centers. The high heterogeneity found demonstrates uncertainty in asthma prevalence in Brazil and highlights the need for clearer protocols addressing the multifactorial nature of the disease.

**Key message**

• This meta-analysis did not allow us to define a consistent geographic pattern of asthma prevalence in Brazil, even when subdivided by region.

• The cartographic representations helped to identify spatial inequalities regarding the prevalence of asthma and showed that most studies carried out in Brazil are in capitals and coastal cities.

• The lack of detailed asthma data in smaller cities can highlight a lack of scientific, political, and investment interest and serves as a warning so that other regions of the world do not present this same scenario.

• State policies need to take into account the multifactorial nature of the disease, considering cultural, social, economic, climatic and geographic differences, in addition to environmental aggression.

Alt-text: Unlabelled box

## Introduction

Asthma is a chronic inflammatory disease of the airways common in young people, clinically characterized by recurrent episodes of wheezing, shortness of breath, and cough.^1^, ^2^, ^3^, ^4^, ^5^ It is considered a global health problem, affecting around 339 million people (Brazil approximately 20 million).^5^ In addition to the respiratory component, there are reports that asthmatic adolescents can compromise their quality of life in general, including difficulty in practicing physical activity and repercussions on mental health.^6^, ^7^, ^8^, ^9^

The need to obtain reliable data in response to the growing increase in the prevalence of asthma worldwide, especially in the early 1990s, demanded the development of epidemiological studies with international collaboration.^10^, ^11^, ^12^, ^13^, ^14^, ^15^, ^16^ The “International Study of Asthma and Allergies in Childhood (ISAAC)”, validated for use in several languages, stands out in this context.^11^ In Brazil, ISAAC had its first phase completed in 1996 (in seven research centers), and was carried out jointly with 56 countries and 155 centers totaling 463,801 adolescents (13 to 14 years old).^11^ The results of this first phase showed a prevalence of active asthma in adolescents worldwide ranging between 1.8 and 36.7 % (Brazil = 19.5 %).^12^^,^^13^

Seven years after the conclusion of Phase I, phase III occurred,^14^ when the number of participating centers increased significantly (233 centers from 97 countries). Worldwide, there was a slight increase in the average prevalence of current asthma among adolescents (average annual increase of 0.06 %).^15^ In Brazil, there was an increase in the number of participating centers and adolescents interviewed (represented by all regions of the country) with the prevalence of asthma reaching 19.0 % (ranging from 11.8 to 30.5 %).^16^ After the creation of ISAAC, its questions have been used in several epidemiological studies around the world.^17^, ^18^, ^19^, ^20^, ^21^

In Brazil, three large national school-based studies that were collected between 2011 and 2014 stand out.^20^, ^21^, ^22^ It is noteworthy that most of the studies found were collected >10 years ago and focused on larger cities, mainly capitals whose majority are located on the coast or in subtropical regions in the states of the South/Southeast region.^20^, ^21^, ^22^, ^23^, ^24^, ^25^, ^26^, ^27^, ^28^, ^29^

Due to its high prevalence, asthma attracts great interest from the scientific community,^10^, ^11^, ^12^, ^13^, ^14^, ^15^, ^16^ however, despite current knowledge about its distribution in different regions of the world and its multifactorial nature (genetics, socioeconomic, behavioral, demographic, dietary, environmental, regional and health),^23^^,^^30^, ^31^, ^32^, ^33^, ^34^, ^35^ the cause of the wide variation found in its prevalence is still unclear. In this sense, Brazil presents a very diverse territory in environmental, socioeconomic, and geographic terms and few studies have explored regional disparities.^20^, ^21^, ^22^ This may compromise a more in-depth analysis of the data and the direction of state policies that may prioritize smaller cities and capitals, without taking into account environmental and regional issues and the multifactorial nature of the disease.^30^, ^31^, ^32^, ^33^, ^34^, ^35^

Most studies on the prevalence of asthma seem to be concentrated in regions of greater economic interest in large cities.^20^, ^21^, ^22^ This fact can limit broader interpretations and make it difficult to plan to combat the disease in a more equitable and universal way. Brazil is a country with large territorial dimensions and there is no detailed data on possible geographic inequalities in its territory. This study carries out a systematic review with meta-analysis to verify the prevalence of asthma in Brazilian adolescents and its geographic pattern. During this study, important questions were raised to answer the present study's objective, such as: (1) Can this study define a consistent pattern of asthma in Brazilian territory? (2) Have current studies on asthma taken climate factors into account? (3) Where are most studies located?

## Material and methods

### Protocol and registration

The systematic review was conducted in accordance with the Preferred Reporting Items for Systematic Reviews and Meta-Analyses (PRISMA)^36^ recommendations. The review protocol was registered in the International Prospective Register of Systematic Reviews (PROSPERO), under registration CRD42023427988.

### Search strategy

The bibliographic search included articles published between 2013 and 2023 and listed in the following databases: Pubmed, Chocrane, LILACS, Scientific Electronic Library Online (SciELO), and SCOPUS. A search was carried out using the PECO strategy regarding the concepts: *P* = Population (population: adolescents in Brazil), *E* = Exposure (exposure: asthma), C = Comparator (control: none) and *O* = Outcomes (outcome: none), as recommended by The Cochrane Handbook for Systematic Reviews of Interventions.^37^ The search terms and the queries applied are presented in Table 1.

**Table 1:** Search strategy in electronic databases.

Data base	Search strategy
PubMed	"Adolescent"[Mesh] OR Adolescen* OR Teen* OR Youth* OR (Adolescent*, Female) OR (Female Adolescent*) OR (Adolescent*, Male) OR (Male Adolescent*) AND "Asthma"[Mesh] OR Asthmas OR (Bronchial Asthma) OR (Asthma, Bronchial) AND "Prevalence"[Mesh] OR Prevalences OR (Period Prevalence) OR (Period Prevalences) OR (Prevalence, Period) OR (Point Prevalence) OR (Point Prevalences) OR (Prevalence, Point) AND "Brazil"[Mesh] OR Brazil* OR Brasil OR (Minas Gerais) OR (São Paulo) OR (Espírito Santo) OR (Rio de Janeiro) OR Bahia OR Pará OR (Mato Grosso) OR (Mato Grosso do Sul) OR Goiás OR (Rio Grande do Sul) OR Ceará OR Pernambuco OR (Santa Catarina) OR Amazonas OR Maranhão OR Tocantins OR Piauí OR Rondônia OR Roraima OR Paraná OR Acre OR Amapá OR Paraíba OR (Rio Grande do Norte) OR Alagoas OR Sergipe OR (Distrito Federal)
SCOPUS	asthma AND prevalence; asthma AND prevalence AND adolescent; asthma AND prevalence AND brazil; asthma AND prevalence AND adolescent AND brazil; asthma AND frequency; asthma AND associated factors; asthma AND risk factor; asthma AND factor intervenient.
LILACS	"Adolescent"[Mesh] OR Adolescen* OR Teen* OR Youth* OR (Adolescent*, Female) OR (Female Adolescent*) OR (Adolescent*, Male) OR (Male Adolescent*) AND "Asthma"[Mesh] OR Asthmas OR (Bronchial Asthma) OR (Asthma, Bronchial) AND "Prevalence"[Mesh] OR Prevalences OR (Period Prevalence) OR (Period Prevalences) OR (Prevalence, Period) OR (Point Prevalence) OR (Point Prevalences) OR (Prevalence, Point) AND "Brazil"[Mesh] OR Brazil* OR Brasil OR (Minas Gerais) OR (São Paulo) OR (Espírito Santo) OR (Rio de Janeiro) OR Bahia OR Pará OR (Mato Grosso) OR (Mato Grosso do Sul) OR Goiás OR (Rio Grande do Sul) OR Ceará OR Pernambuco OR (Santa Catarina) OR Amazonas OR Maranhão OR Tocantins OR Piauí OR Rondônia OR Roraima OR Paraná OR Acre OR Amapá OR Paraíba OR (Rio Grande do Norte) OR Alagoas OR Sergipe OR (Distrito Federal)
SciELO	"Adolescent"[Mesh] OR Adolescen* OR Teen* OR Youth* OR (Adolescent*, Female) OR (Female Adolescent*) OR (Adolescent*, Male) OR (Male Adolescent*)) E ("Asthma"[Mesh] OR Asthmas OR (Bronchial Asthma) OR (Asthma, Bronchial)) E ("Prevalence"[Mesh] OR Prevalences OR (Period Prevalence) OR (Period Prevalences) OR (Prevalence, Period) OR (Point Prevalence) OR (Point Prevalences) OR (Prevalence, Point)) E ("Brazil"[Mesh] OR Brazil* OR Brasil OR (Minas Gerais) OR (São Paulo) OR (Espirito Santo) OR (Rio de Janeiro) OR Bahia OR Pará OR (Mato Grosso) OR (Mato Grosso do Sul) OR Goiás OR (Rio Grande do Sul) OR Ceará OR Pernambuco OR (Santa Catarina) OR Amazonas OR Maranhão OR Tocantins OR Piauí OR Rondônia OR Roraima OR Paraná OR Acre OR Amapá OR Paraíba OR (Rio Grande do Norte) OR Alagoas OR Sergipe OR (Distrito Federal)

### Eligibility criteria

Articles that presented a measure relative to the prevalence of asthma in adolescents of both sexes available in full in English were included. Studies that were published between 2013 and December 2023 were included. Regarding data collection, studies that occurred from 2012 onwards or that were collected before, but ended in 2012, were included. Exclusion criteria for the studies were: nonoriginal research (e.g. systematic or literature reviews) or not published in a final peer-reviewed journal (e.g. letters to the editor, editorials, comments, and presentations at conferences, congresses, or seminars), as well as dissertations or course completion work.

### Selection of studies

**Figure 1:**
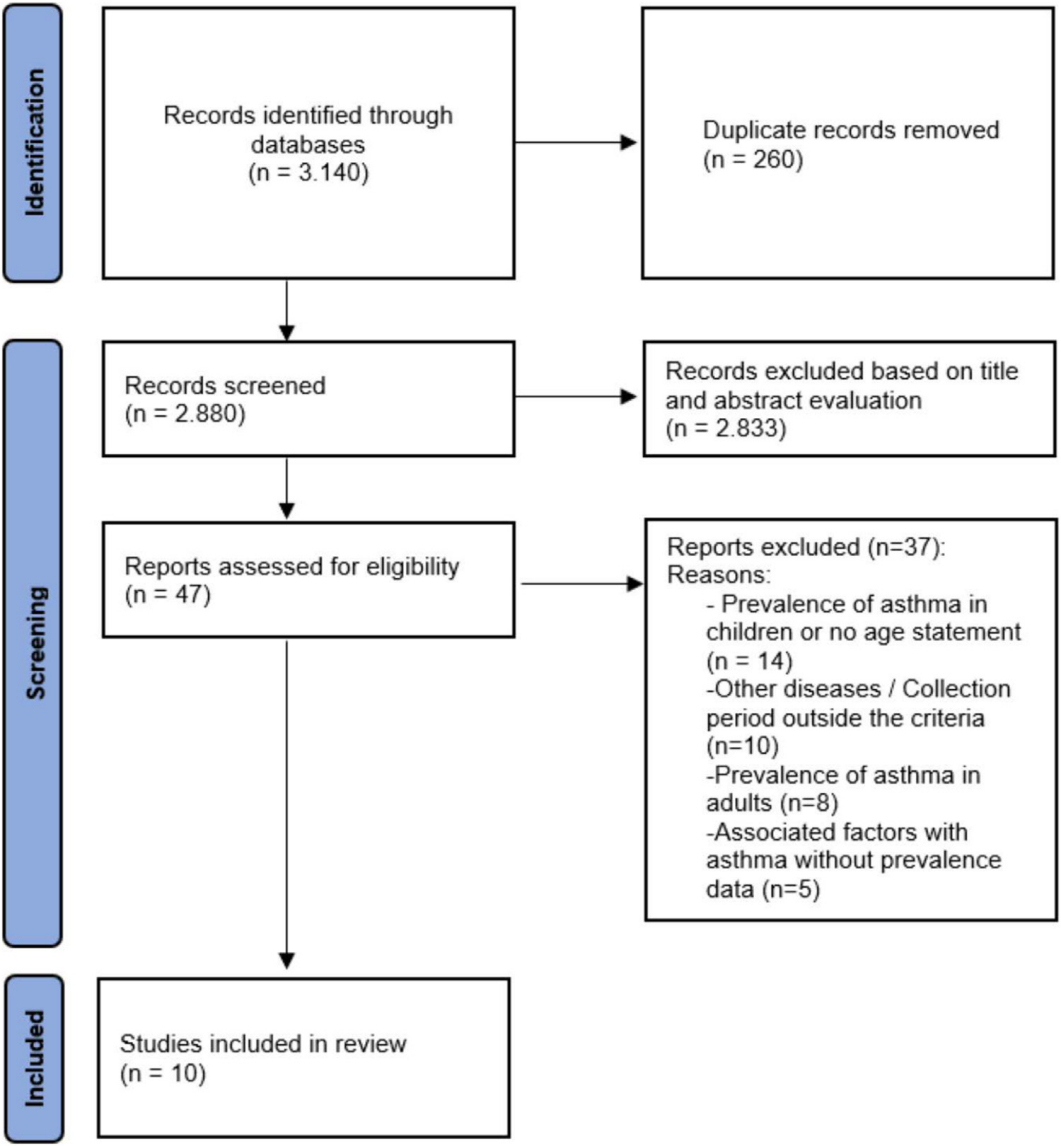
Study selection flowchart.

Two independent reviewers (M.C.P.O. and E. K. S. M.) selected studies in two steps: (1) title and abstract screening; (2) and full-text reading. Eligibility for inclusion in the two-rater review was coded as ‘yes’, ‘no’ or ‘maybe’. Duplicate studies were excluded. Disagreements between reviewers were resolved by consensus and, when necessary, the third researcher (MAVCJ) was consulted to make the final decision. Figure 1 shows the flowchart containing the stages of the process of searching and selecting articles included in this systematic review. Database provided by the authors were also consulted when necessary.

### Data extraction

Two reviewers independently (M.C.P.O. and E. K. S. M.) extracted data from published articles, using a standard form from the Cochrane Collaboration model.^37^ Studies that met the eligibility criteria were entered into a Microsoft Excel software spreadsheet (Microsoft Corporation, WA, USA) containing the following information: authors, year of publication, region, age, year of collection, sample size, information about sex, how the outcome was assessed (data collected) and whether there was funding and conflict of interest. Additional information was requested by email (one study). Some data needed to be calculated from the results shown in the tables and figures (four studies).

### Assessment of methodological quality: risk of bias

To assess the risk of bias, the Joanna Brigg Institute (JBI) tool was used (prevalence studies).^38^ Each item can be answered with “yes”, “no”, “unclear” and “not applicable”, with the answer “yes” suggesting a good-quality resource of the study and the answer “no” of a poor-quality resource. The evaluation was carried out independently by the two evaluators. When disagreement occurred, the authors discussed their reasons, and the final decision was made by consensus. Methodological quality was categorized as “Low” when the study obtained up to four “Yes” answers to the items evaluated; “Moderate” when the study obtained five or seven “Yes” responses; and “High” when the study reached eight or more “Yes” responses. This classification was included to facilitate interpretation and is permitted by the guidance JBI manual for assessing the methodological quality of prevalence studies.^38^

### Statistical analysis

Analyses and graphs were performed using R-studio statistical software, version 4.1.3 (R-studio Inc). A meta-analysis of proportions was carried out with a random effect model using the DerSimonian and Laird estimator to calculate Tau-square (τ²).^39^ Heterogeneity was assessed using the Higgins inconsistency index (I²).^40^ The Freeman–Tukey double-arcsine transformation method was used to stabilize the variance of each study's proportion.^41^ To stipulate the weight of the studies included in the analysis, the effect size of each study was weighted using the inverse variance method, calculating the estimate based on the inverse proportion of the study's variance.^41^ The 95 % confidence intervals (95 % CI) were calculated using the Clopper-Pearson method. To evaluate possible causes of heterogeneity, subgroup analysis was analysed according to region.

In the persistence of heterogeneity, possible confounding factors were explored according to the moderating variable. In this way, subgroup analysis was performed, dividing the meta-analysis groups according to the category of regions of Brazil used in the studies.

A sensitivity analysis was performed to assess whether studies classified as high risk of bias would influence the effect size. The graphical assessment of the existence of publication bias was carried out using the funnel plot, in addition to the Egger test, to assess the existence of asymmetry.

## Results

### Identification and selection of studies

After removing duplicates and analyzing titles and abstracts, of the 3.140 titles, 47 articles remained to be reviewed and analyzed in full, of which 10 were considered relevant for this review. An in-depth analysis defined the exclusion of 37 publications: 1- Prevalence of asthma in children and schoolchildren, 2- Other diseases / Collection period outside the criteria, 3- Prevalence of asthma in adults, and 4- Factors associated with asthma (Figure 1).

### Characteristics of the studies

Table 2 summarizes the description of the characteristics of the included studies. All studies used the questions from the ISAAC instrument to assess active asthma (‘‘In the last 12 months, have you had wheezing?’’), severe asthma (those adolescents classified as having active asthma who reported chest wheezing that was strong to the point of affecting speech), and diagnosed asthma (‘‘Have you ever had asthma in your life?’’). The years of publication ranged from 2014 to 2021. The age of the adolescents ranged between 10 and 19 years old. Regarding data collection, six publications were collected before 2015, one in 2016–2018, one without information, and two in 2018. The National Adolescent School-based Health Survey (PeNSE 2012)^20^ and Cardiovascular Risks in Adolescents (ERICA)^21^ studies did not report the names of cities that were not capitals.

**Table 2:** Characteristics of the included studies.

Authors, year	Region	Age (Years)	Year of collection	n	Sex ♂ / ♀	Data collected	Funding	Conflict of interest
Barreto et al.^20^	N/NE/CW/SE/S	13–15	2012	61.145^d^	47,6 / 52,4^e^	AT/DA	N	ND
Correia et al.^23^	NE	13–14	2014	1.591	49,7 / 50,3	AT/SA/DA	NI	N
Kuschnir et al.^21^^,^^a^	N/NE/CW/SE/S	12–17	2013–2014	55.628^d^	44,7 / 55,3^e^	AT/DA	Y	N
Medeiros et al.^24^	NE	13–14	NI	3.268	45,9 / 54,1	AT/SA/DA	NI	N
Neto et al.^25^	NE	13–14	2018	1.132	45,5 / 54,5	AT/SA/DA	NI	N
Oliveira et al.^26^^,^^c^	S	10–19	2018^b^	187	35,8 / 64,2	AT/DA	Y	N
Schuh et al.^27^	S	10–18	2013	964	47,9 / 52,1	AT/AD	NI	NI
Solé et al.^22^	N/NE/S/SE	13–14	2011–2012^b^	20.099	NI / NI	AT/SA/DA	Y	N
Urrutia-Pereira et al.^28^	S	13–14	2016–2018	1.058	55,1 / 44,9	AT/SA/DA	N	N
Wilmer et al*.*^29^	S	12–14	2012^b^	2.563	NI / NI	AT/SA/DA	NI	NI

All studies were cross-sectional and classified according to ISAAC.

N, North (for region); NE, Northeast; CW, Central West; SE, Southeast; S, South; AT, Active Asthma; SA, Severe Asthma; DA, Diagnosed Asthma; N, No; Y, Yes; ND, Nothing to declare; NI, Not informed; ♂, Male; ♀, Female.

a The authors inserted the term “crises” in the original ISAAC question.

b Data collected or finalized above 2012 were considered.

c Only population-based study (the other studies were school-based).

d The National Adolescent School-based Health Survey (PeNSE)^20^ and Cardiovascular Risks in Adolescents (ERICA)^21^ studies did not report the names of cities that were not capitals.

e Data taken from total prevalence.

### Risk of bias

The methodological quality of the studies is summarized in Table 3. Two studies presented moderate methodological quality, as they lost points in the detailed description of the subjects researched and because they did not detail whether there were losses in the study. The remaining studies presented high methodological quality.

**Table 3:** Assessment of methodological quality of the studies.

	Joanna Brigg Institute quality assessment tool (prevalence studies)										
Study	Q1	Q2	Q3	Q4	Q5	Q6	Q7	Q8	Q9	Total	Quality
Barreto et al.^20^	Y	Y	Y	NA	Y	Y	Y	Y	N	07	Moderate
Correia et al.^23^	Y	Y	Y	Y	Y	Y	Y	Y	Y	09	High
Kuschnir et al.^21^	Y	Y	Y	NA	Y	Y	Y	Y	N	07	Moderate
Medeiros et al.^24^	Y	Y	Y	Y	Y	Y	Y	Y	N	08	High
Neto et al.^25^	Y	Y	Y	Y	Y	Y	Y	Y	Y	09	High
Oliveira et al.^26^	Y	Y	Y	Y	Y	Y	Y	Y	N	08	High
Schuh et al.^27^	Y	Y	Y	Y	Y	Y	Y	Y	N	08	High
Solé et al.^22^	Y	Y	Y	Y	Y	Y	Y	Y	N	08	High
Urrutia-Pereira et al.^28^	Y	Y	Y	Y	Y	Y	Y	Y	N	08	High
Wilmer et al.^29^	Y	Y	Y	Y	Y	Y	Y	Y	Y	09	High

Q1: Was the sample frame appropriate to address the target population?; Q2: Were study participants recruited in an appropriate way?; Q3: Was the sample size adequate?; Q4: Were the study subjects and setting described in detail?; Q5: Was data analysis conducted with sufficient coverage of the identified sample?; Q6: Were valid methods used for the identification of the condition?; Q7: Was the condition measured in a standard, reliable way for all participants?; Q8: Was there appropriate statistical analysis?; Q9: Was the response rate adequate, and if not, was the low response rate managed appropriately?

Y, Yes; N, No; NC, Unclear; NA, Not Applicable.

### Meta-analysis, geographic pattern, and level of evidence

**Figure 2:**
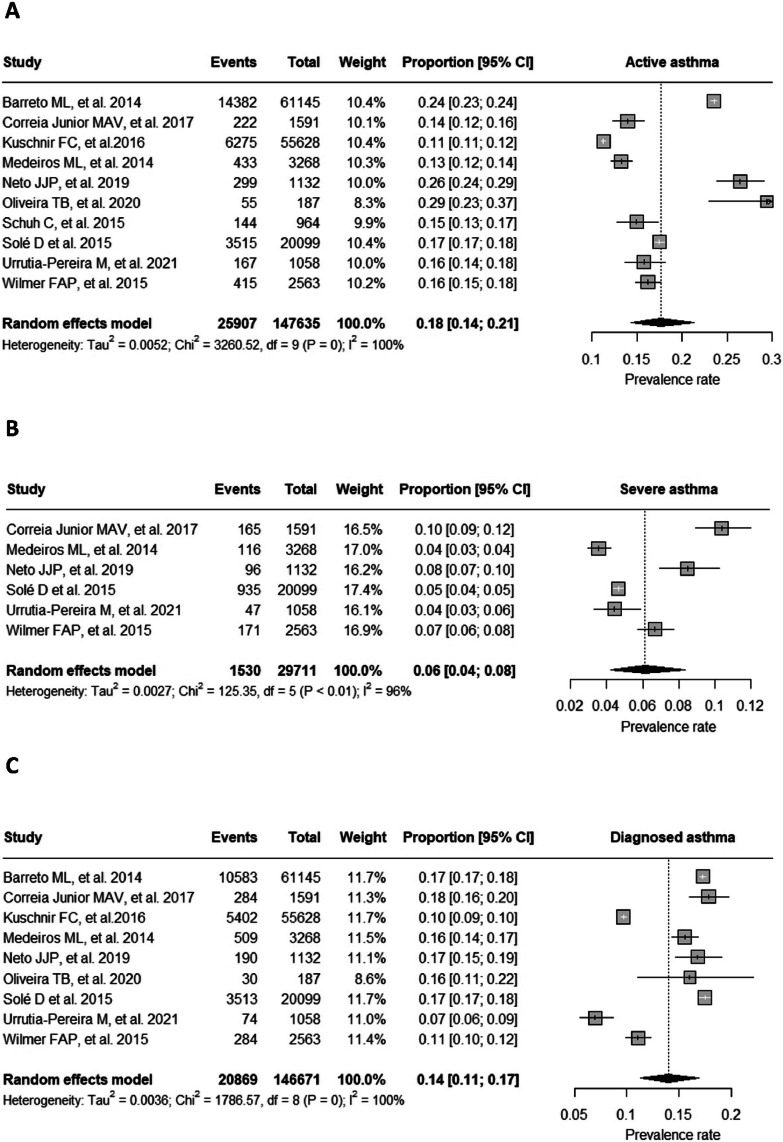
Forest Plot graph of active (A), severe (B) and diagnosed asthma (C). In relation to the ERICA^21^ and PeNSE^20^ studies, this meta-analysis used data extracted directly from the table published by the authors that showed the prevalence of active asthma by municipality.

The prevalence of active asthma, severe asthma and diagnosed asthma in Brazil was 18 %, 6 % and 14 %, respectively (Figure 2A, B, and C). The effects of individual studies showed a high level of heterogeneity.

**Figure 3:**
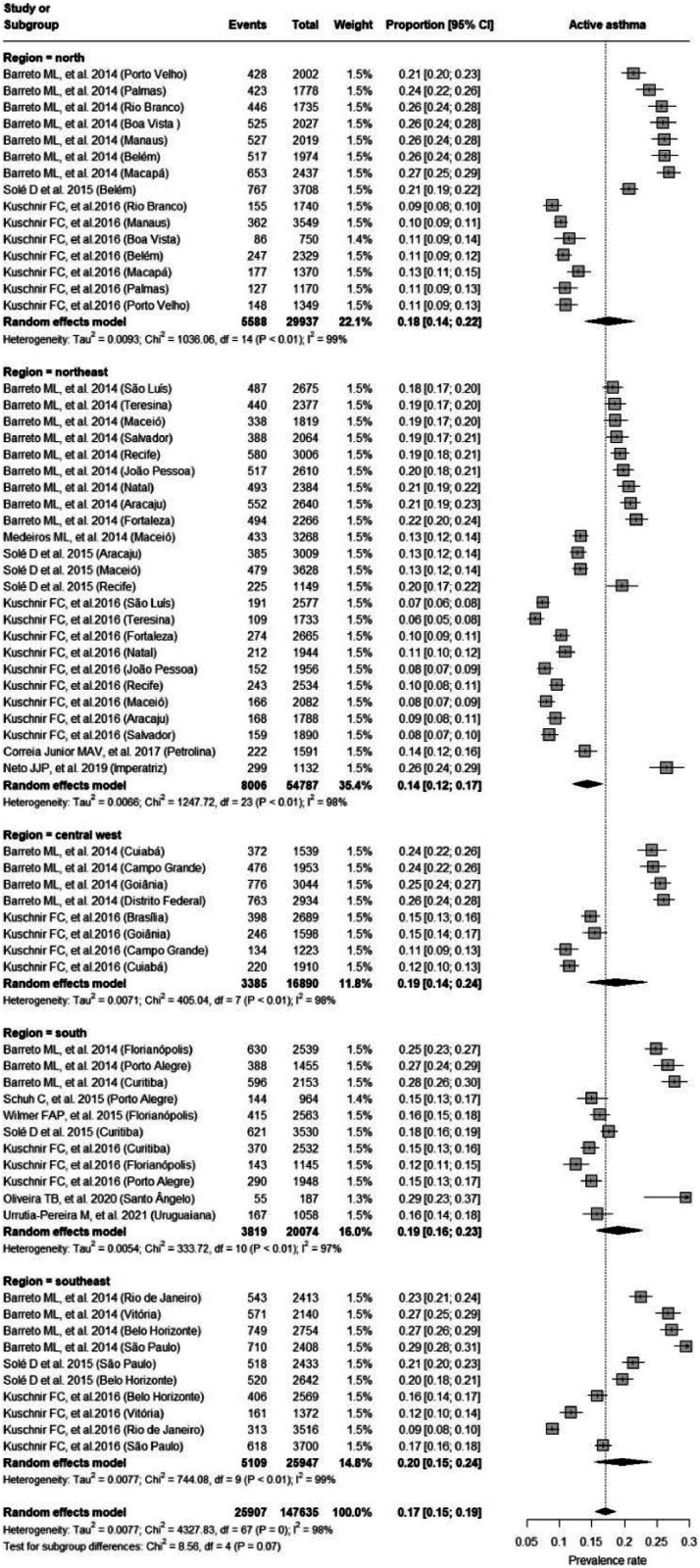
Forest Plot of active asthma by regions in Brazil.

**Figure 4:**
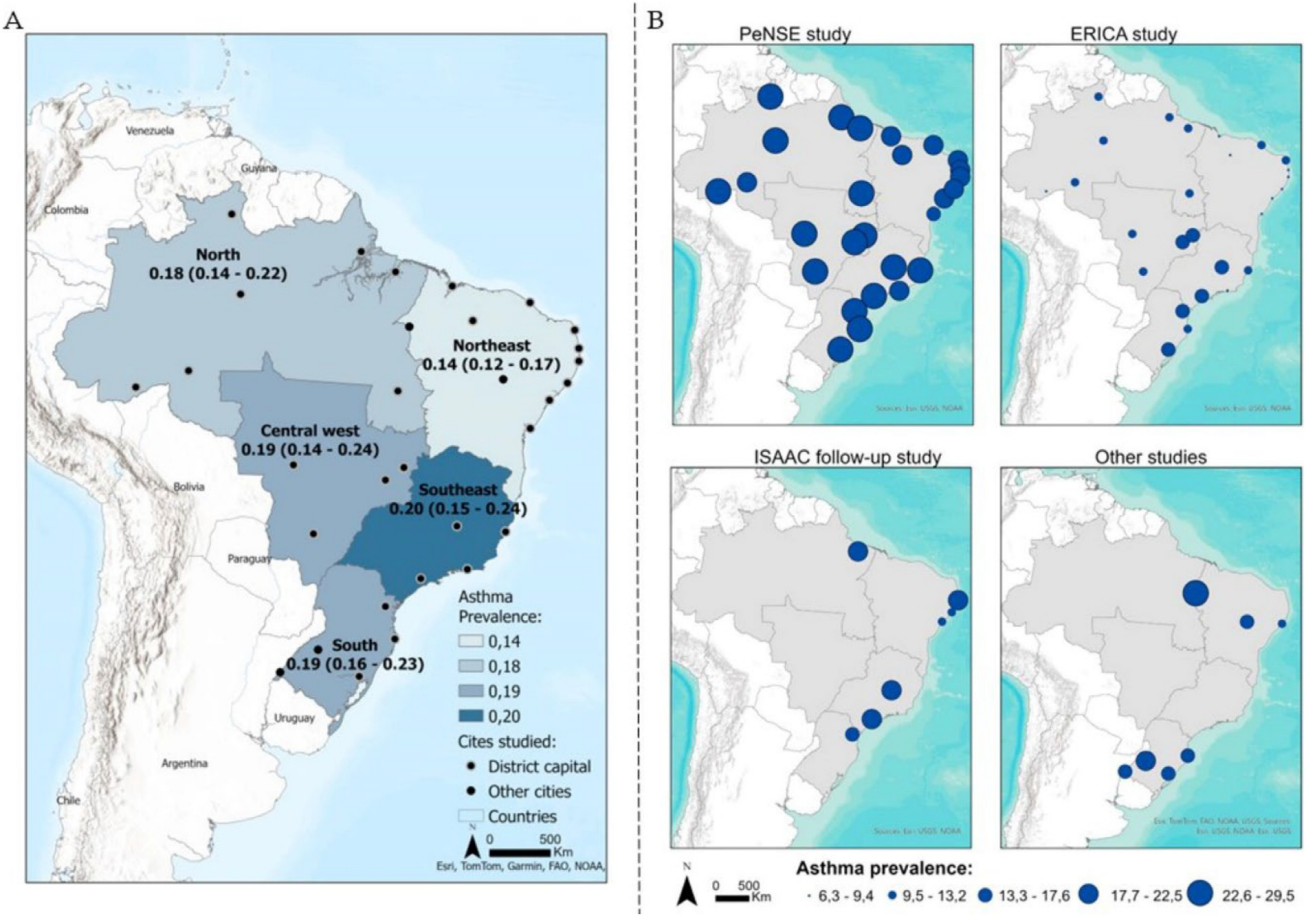
Geographic map representing the prevalence of active asthma in Brazil and its regions (A) and prevalence of asthma according to studies (B).

To explore heterogeneity, subgroup analysis was performed, separating the cities according to the different regions of Brazil (north, northeast, central-west, south, and southeast). The Northeast region had a lower prevalence, *p* < 0.001 (Figure 3, Figure 4A). There was no difference between regions in relation to severe asthma and diagnosed asthma (supplementary materials 1 and 2). A total of 31 cities were evaluated, of which 27 (87.1 %) were capitals of the federative units (Figure 4A). Figure 4B shows the prevalence of active asthma according to the studies and their dimensions on the cartographic map. The Köppen climate classification^42^ of Brazil is present in the supplementary material 3.

According to the GRADE assessment (Table 4) the quality of evidence was very low due to high heterogeneity (inconsistency) and wide CIs (imprecision) in the estimates obtained.

**Table 4:** Quality of evidence using the GRADE approach.

Certainty assessment							Nº of patients		Effect	Certainty	Importance
No. of studies	Study design	Risk of bias	Inconsistency	Indirectness	Imprecision	Other considerations	Adolescent	Asthma	Absolute (95 %CI)		
10	observational studies	not serious^a^	very serious^b^	not serious	very serious	none	147.635	25.907	prevalence **0.18 % higher** (0.14 higher to 0.21 higher)	⨁○○○ Very low	Important

CI, Confidence interval.

a The studies presented a low risk of bias regarding methodological quality for prevalence research.

b I² = 100 %.

### Reporting bias and sensitive analysis

Analysis of the active asthma funnel plot demonstrated that studies with smaller samples, and consequently larger standard errors, were distributed symmetrically in the widest part of the funnel plot for both sides of the estimate, suggesting the absence of publication bias (Supplementary material 4). Likewise, the Egger test confirmed the absence of publication bias (*p* = 0.955).

Sensitivity analysis was performed to verify the influence of the studies by Barreto et al.^20^, and Kuschinir et al.^21^, as they were classified as having a moderate risk of bias. The overall meta-analysis estimates for active, severe, or diagnosed asthma did not change significantly after sensitivity analysis.

## Discussion

This systematic review with meta-analysis found a high proportion of Brazilian adolescents who reported active asthma (18 %), with the northeast region (14 %) having the lowest prevalence. These results are generally higher than the global prevalence of asthma (14.1 %), as well as those found in North America (21.6 %), in contrast to India (7 %), Africa (14 %), Western Europe (14.3 %) and Eastern Europe (9.7 %).^10^ The prevalence of severe asthma and diagnosed asthma was 6 % and 14 %, respectively, with no statistically significant differences between regions. Most of the municipalities studied are located in capitals and large urban centers. Due to the high heterogeneity observed between studies, it is difficult to identify a geographic pattern for asthma prevalence.

School-based epidemiological studies on the prevalence of asthma in adolescents are important to identify the distribution and scenario of the disease, in addition to providing information so that state policies can be implemented based on scientific evidence.^10^^,^^13^^,^^20^, ^21^, ^22^ The compilation of data from previous studies with a similar methodology synthesized in a meta-analysis can provide a strategic vision of where to take a more careful look and consequently direct more investment with higher quality.^13^^,^^43^ Brazil has around 5500 municipalities represented by 27 federative units (26 state capitals and one federal capital). The present results show that all 27 capitals were represented in this systematic review of a total of 31 municipalities. In this sense, the panorama shown in this research presents that most studies were carried out in capitals and large urban centers, which may present a bias regarding the population studied, showing the need to understand other contexts in Brazil, considering the high spatial disparities and the multifactorial nature of the disease.

Another fact that draws attention is the wide variation found in asthma prevalence, even when evaluated in the same city. In Brazil, three studies^20^, ^21^, ^22^ stand out for having records in all regions of the territory. National Adolescent School-based Health Survey (PeNSE 2012)^20^ and an ISAAC follow-up study (2003–2012)^22^ data collection were developed in 2012 and showed a prevalence of active asthma of 23.2 % and 17.5 % for ages 13 to 14 years and 13 to 15 years, respectively. A lower prevalence (13.1 %) was reported by the Study of Cardiovascular Risks in Adolescents (ERICA)^21^ when investigating adolescents aged 12 to 17 (collection years 2013 and 2014). The authors^21^ presented two hypotheses to justify this lower prevalence. The first was in relation to the broader age range, however, the researchers^21^ explain that there was no significant change in prevalence when adjusted for age. A second fact concerns a modification of the ISAAC questionnaire question. The words “crises of” were added before the original question “wheezing in the last 12 months”, which may have reduced the sensitivity of the ISAAC question and influenced the results with lower prevalence.

Considering the disparities in the prevalence estimate and risk of bias associated with the ERICA^21^ and PeNSE^20^ studies, the authors performed a sensitivity analysis removing these two studies from the meta-analysis, and no significant differences in prevalence were found. In this sense, it was decided to maintain both studies. Other studies in Brazil report prevalence rates that ranged from 6 % in Teresina in the Northeast^21^ to 29 % in São Paulo and Santo Ângelo in the Brazilian southeast and south.^20^ Although the Northeast region has the lowest prevalence of asthma (14 %), occurrence rates across cities varied between 6 % and 26 % (Teresina and Imperatriz, respectively). These variations also occurred in the North region (18 %) with values ​​ranging from 9 % to 27 % (Rio Branco and Macapá, respectively), the Central West region (19 %) with a variation between 11 % and 26 % (Campo Grande and Distrito Federal, respectively), the Southeast region (20 %) with a variation between 9 % and 29 % (Rio de Janeiro and São Paulo, respectively) and the South region (19 %) with a variation between 12 % and 29 % (Florianopolis and Santo Ângelo, respectively).

The weak representation of studies in some areas of the country stands out, notably, a single study developed in a semi-arid climate (prevalence of active asthma 14 %) was identified.^23^ The literature has reported lower prevalences in dry climates and describes a possible association with the proliferation of house dust mites, which cannot survive in environments with low humidity and are one of the main determinants of allergic sensitization.^35^^,^^44^^,^^45^ Other cultural, genetic, environmental, social, and economic factors, exposure to smoke, drinks, and even the season (since the period before spring can lead to the manifestation of allergic asthma attacks, due to greater exposure to pollens of plants, in addition to regions that present a long period with low air humidity) must be taken into consideration when analyzing the conditions that explain the inequalities in asthma prevalence.^20^, ^21^, ^22^^,^^34^^,^^45^, ^46^, ^47^, ^48^

Due to the importance of data for public health, the current study encourages researchers in epidemiology and immuno-allergic diseases to use instruments that are already validated and accepted worldwide so that their data can be compared and inserted into decision-making. The results presented here show the difficulty in identifying a consolidated prevalence pattern given the large variation in results and confirmed by the high heterogeneity in the meta-analysis. In the same sense, the Global Strategy for the Management and Prevention of Asthma (GINA)^5^ has highlighted the global difficulty in controlling the disease. Since 2011, the Brazilian government has provided free medication for asthma,^49^ with the potential to reduce symptoms, control the disease and, consequently, the population's healthcare costs. However, it seems that the reach of these programs is not adequate across the entire territory and scientific data is concentrated in large urban centers.^35^ Another issue is that the re-evaluation of these programs occurs in an isolated and infrequent manner, without a national organization.^50^ Other studies could better explore the socioeconomic context of each individual, with a focus on housing conditions and air pollution (inside and outside homes). Another line of investigation could be related to the period that some Brazilian cities are exposed to low air humidity and the relationship with allergies caused by dust mites.

Future research may consider incorporating artificial intelligence and machine learning tools to provide more accurate and timely epidemiological data through the usage of big data. Previous initiatives utilizing these resources estimated more accurate results with less heterogeneity.^51^ Even so, the information about the severity of asthma present in the ISAAC questionnaire can generate important evidence in epidemiological studies regarding the lack of control of the disease and the direction of investment, whether in guidance or in state policies.^5^^,^^52^

Regarding severe asthma, the data available for analysis were more limited (only six studies evaluated eleven districts out of a total of ten that evaluated 31 cities for active asthma). In this case, only one city was evaluated in the North region (Belém) and none in the Central West region. The prevalence of asthma severity in Brazil was 6 %, which varied between 4 % (Maceio, Aracaju, Uruguaiana, São Paulo) and 10 % (Petrolina), however, it is noteworthy that the Brazilian study registered the highest asthma severity (10 %) and a relatively low prevalence of active asthma (14 %).^23^ As in the present study, the authors^23^ describe the need for research in regions that are not large centers and highlight the possibility of a greater lack of assistance from the state in these locations, with greater difficulty in distributing medicines to control the disease, difficulty in finding specialized assistance and consequently greater episodes of severe asthma. From the interpretation of the results of this meta-analysis, the authors can infer that asthma, at least on the part of the professionals who diagnose the disease, does not seem to be neglected, since the frequency of diagnosed asthma was slightly lower (14 %) than that of active asthma (18 %). However, this is no guarantee that patients are being treated appropriately.

The existence of possible uncontrolled confounding factors may have been the cause of the wide variety in prevalence found, even when the same city was seen by different authors. Brazil is a large country and even when divided into regions, it presents very different characteristics in terms of geography, socioeconomic, cultural, and demographic characteristics. As possible limitations of this review, the authors can mention that the geographic pattern was not able to identify a consolidated model of the prevalence of active, severe, or diagnosed asthma, even when evaluated by regions. Even so, this was the first systematic review that alerts us to the need to include more substantial data in the collections, especially regarding the time of year collected, climatic and geographic characteristics, air quality conditions, in addition to social and economic characteristics. Another relevant question would be to know the public expenditure related to medication and issues relating to hospitalizations. This study also warns that publications on asthma on a national basis detail their data collected on prevalence in non-capital cities.

The highest points of this discussion are: the meta-analysis did not allow defining a consistent geographic pattern of asthma prevalence, research has not valued issues relating to humidity, air quality and effects of environmental aggression and cartographic representations have helped to identify spatial inequalities, in addition to showing that the majority of Brazilian studies are in capitals and coastal cities. Public calls with funding and national organizations aimed at studying the prevalence and risk factors of immuno-allergic diseases that consider cultural, social, economic, environmental, climatic, and geographic differences in sample planning could be more assertive strategies, to better target assistance. This method, together with the free distribution of drug therapy (already existing in Brazil) and non-drug therapy (e.g. stimulation of physical activity), as described in the guidelines, could help in combating and directing resources in a decentralized way based on the understanding of the data provided by epidemiological surveillance.

## Conclusion

This study proposes the need for better quality data able to support orientated policies addressing the nuances of asthma prevalence in young and its impacts. Higher quality information systems can support the identification of cities with higher incidence and help control the disease, alerting patients to take environmental precautions against aeroallergens such as fungi, mites and pollens, or even warning about smoke related to wildfires, exposure to wind-blown dust, dry weather and periods of greater risk of respiratory infections, as well as informing society and the government about the need for hospital beds and professionals interested in the study of asthma.

Prevalence needs to be assessed periodically, especially under conditions of climate change. In this sense, studies are needed that explore the geographic characteristics and socioeconomic conditions of each region, emphasizing the relationship between the macrosystem and the people. Based on robust data and population representation, each city will be able to allocate resources and structure for health care, choosing whether the priority will be education, prevention, or treatment of the disease at the outpatient or hospital levels.

## Conflicts of interest

The authors declare no conflicts of interest.

## Appendix Supplementary materials

Image, application 1
