# Supplementary material for: Geographic pattern of asthma prevalence in Brazilian adolescents: a systematic review with meta-analysis
Source: J Pediatr (Rio J). 2025 Feb 14;101(3):312–23. doi: 10.1016/j.jped.2024.12.004 (PMC12039388; doi:10.1016/j.jped.2024.12.004)
Supplement: Supplementary file 1 [file mmc1.docx]

**JPED-D-24-00544_Supplementary material**


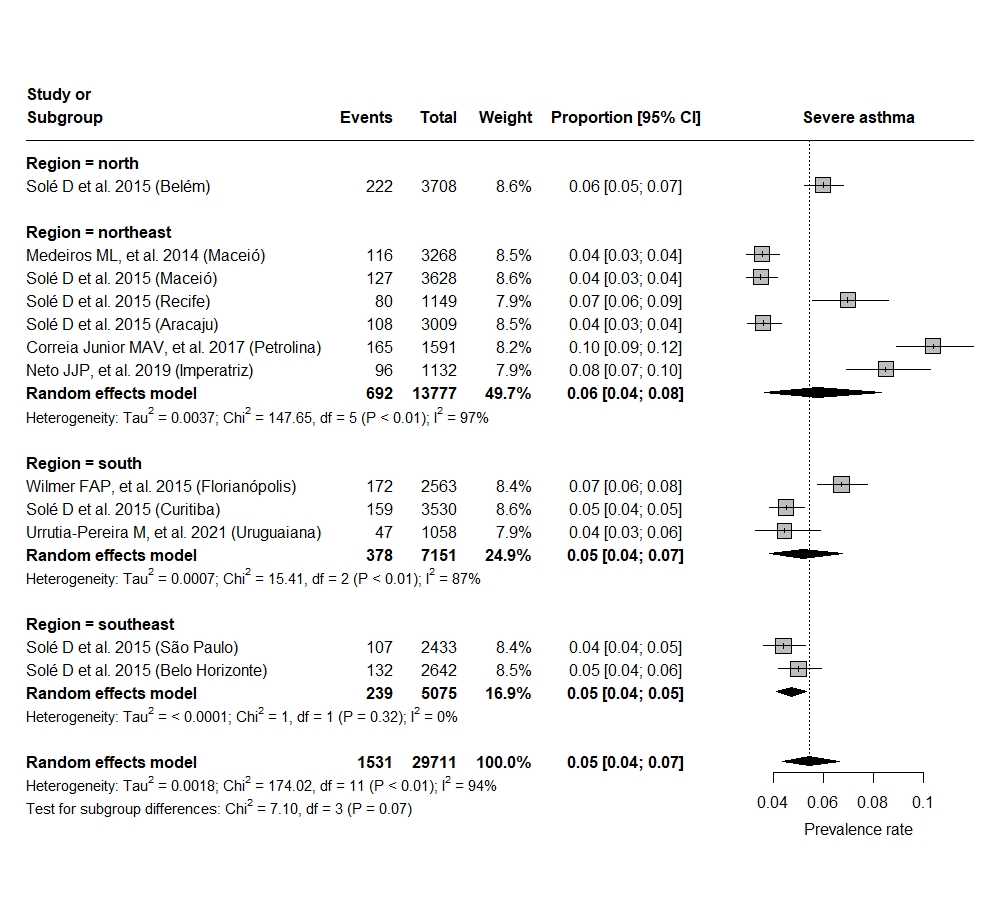


**Figure Supplementary 1.** Forest Plot graph of severe asthma by regions of Brazil.


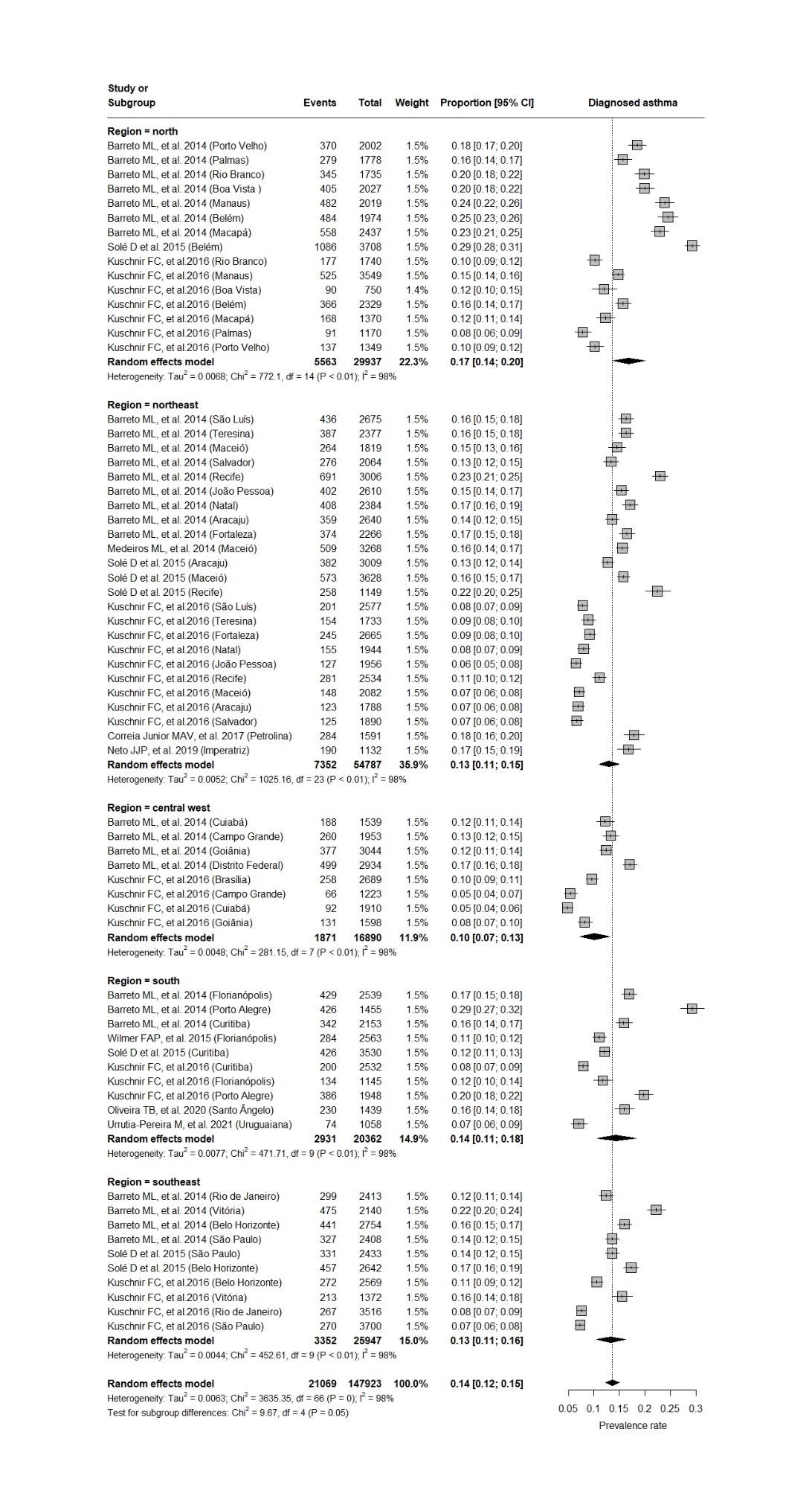


**Figure Supplementary 2.** Forest Plot graph of asthma diagnosed by regions of Brazil.


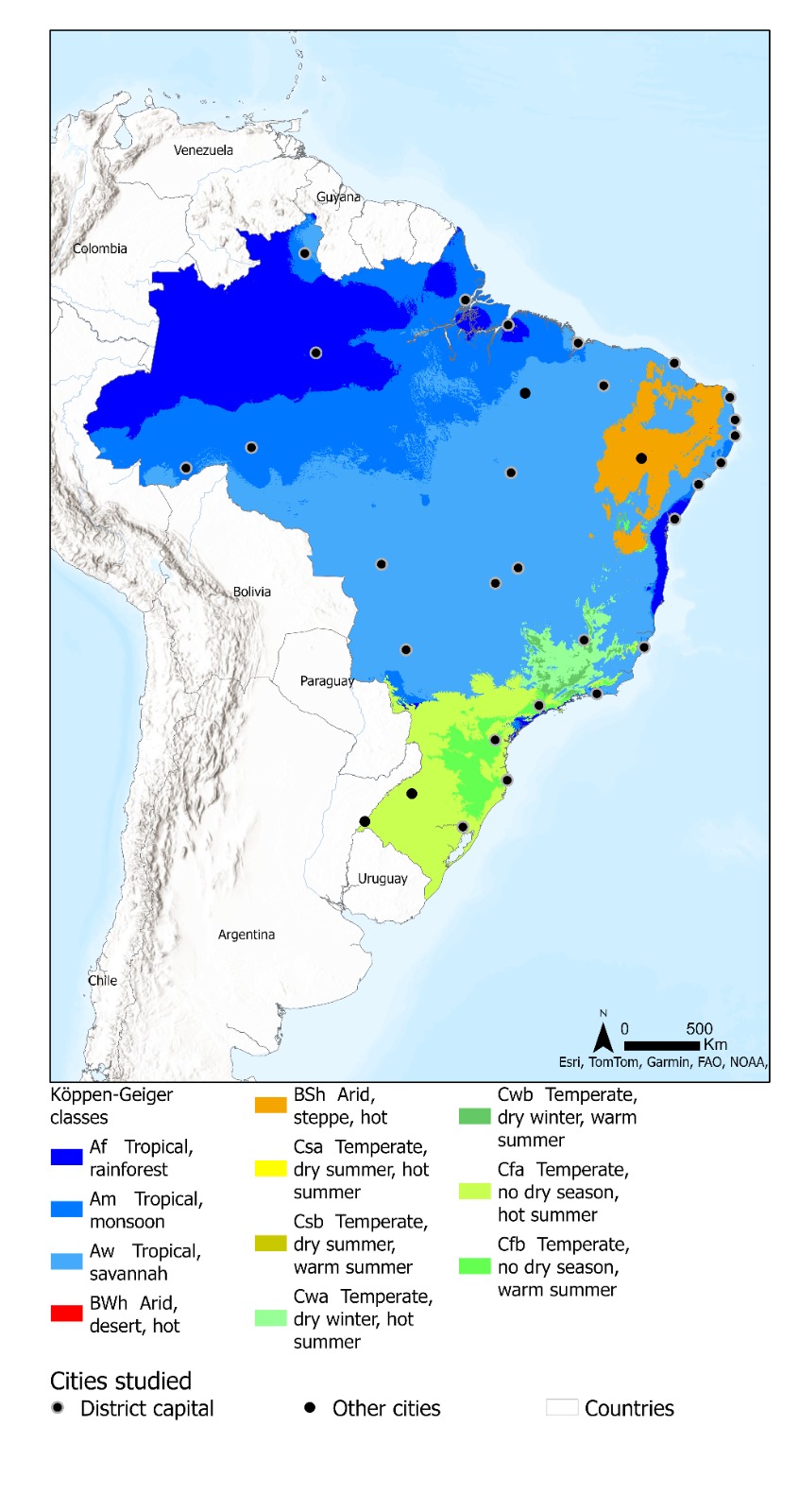


**Figure Supplementary 3.** Map of Brazil representing cities according to the Köppen climate classification^42^


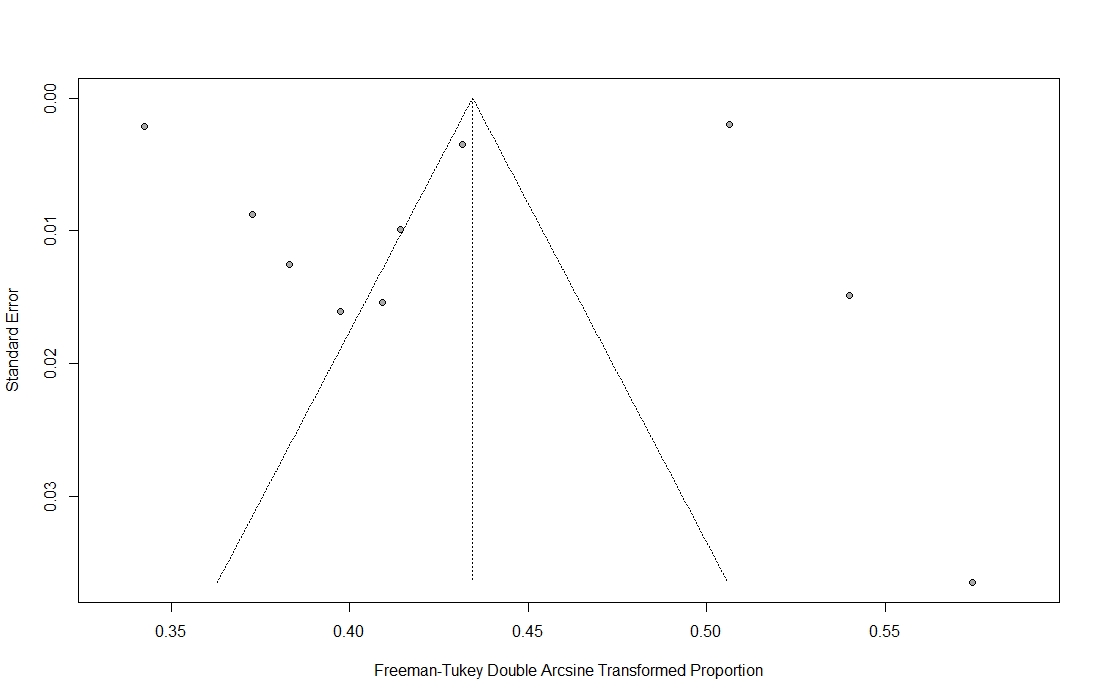


**Figure Supplementary 4.** Asthma funnel plot showing the existence of studies with a higher standard error of the observed effect.
